# Vitamin B_12_ Attenuates Acute Pancreatitis by Suppressing Oxidative Stress and Improving Mitochondria Dysfunction via CBS/SIRT1 Pathway

**DOI:** 10.1155/2021/7936316

**Published:** 2021-12-09

**Authors:** Jiyan Yuan, Zeliang Wei, Guang Xin, Xubao Liu, Zongguang Zhou, Yi Zhang, Xiuxian Yu, Chengyu Wan, Qingqiu Chen, Weiyu Zhao, Xueling Wang, Yuman Dong, Zhen Chen, Xiaoting Chen, Hai Niu, Wen Huang

**Affiliations:** ^1^Laboratory of Ethnopharmacology, West China School of Medicine, West China Hospital, Sichuan University, Chengdu, Sichuan, China; ^2^Department of Dermatology, West China Hospital, Sichuan University, Chengdu, Sichuan, China; ^3^Department of Pancreatic Surgery, West China Hospital of Sichuan University, Chengdu, Sichuan, China; ^4^Department of Gastrointestinal Surgery and Laboratory of Digestive Surgery, West China Hospital, Sichuan University, Chengdu, China; ^5^Research Core Facility of West China Hospital, Sichuan University, Chengdu, Sichuan, China; ^6^Integrated Chinese and Western Medicine Department, West China Hospital, Sichuan University, Chengdu, Sichuan, China; ^7^Animal Experimental Center, West China Hospital, Sichuan University, Chengdu, Sichuan, China

## Abstract

Acute pancreatitis is an inflammatory disorder of the pancreas associated with substantial morbidity and mortality, which is characterized by a rapid depletion of glutathione (GSH). Cysthionine-*β*-synthase (CBS) is a key coenzyme in GSH synthesis, and its deficiency is related to a variety of clinical diseases. However, whether CBS is involved in the pathogenesis of acute pancreatitis remains unclear. First, we found that CBS was downregulated in both *in vivo* and *in vitro* AP models. The pancreatic damage and acinar cell necrosis related to CBS deficiency were significantly improved by VB 12, which stimulated clearance of reactive oxygen species (ROS) by conserving GSH. Furthermore, EX-527 (a specific inhibitor of SIRT1) exposure counteracted the protective effect of VB 12 by promoting oxidative stress and aggravating mitochondrial damage without influencing CBS, indicating that vitamin B_12_ regulates SIRT1 to improve pancreatical damage by activating CBS. In conclusion, we found that VB 12 protected acute pancreatitis associated with oxidative stress via CBS/SIRT1 pathway.

## 1. Introduction

Acute pancreatitis (AP) is an inflammatory disease of exocrine pancreas associated with tissue lesions and necrosis [1, 2]. The disease usually accompanied with systemic inflammatory response syndrome-associated extrapancreatic organ failure and even death [3]. Importantly, no therapeutic agents currently in use could alter the course of the disease. The development of treatments for AP is, therefore, a priority, one strategy for which is to follow leads from complementary laboratory and clinical studies, as here.

Pancreatic glutathione (GSH) depletion is an early feature in the development of acute pancreatitis, and its long-term depletion can exacerbate the severity of disease [4, 5]. Cysteine-*β*-synthase (CBS) is widely distributed in the liver, kidney, and pancreas and is the first (and rate-limiting) enzyme in the GSH synthesis pathway [6, 7]. A recent study showed CBS gene knockdown promotes inflammation and oxidative stress in immortalized human adipose-derived mesenchymal stem cells, enhancing their adipogenic capacity [8]. In addition, the decreased expression of CBS propagates the pathogenesis of ulcerative colitis by exacerbating inflammation-induced intestinal barrier injury [9], implying an important role of CBS in mediating organ damage. However, few studies focus on CBS in acute pancreatitis.

GSH is the principal intracellular antioxidant, which may act directly by scavenging reactive oxygen and nitrogen species or indirectly by supporting enzymatic activity as a cofactor. Depletion of GSH is difficult to maintain the redox balance, leading to a large accumulation of reactive oxygen species (ROS) in cells. Mitochondria are the main source of ROS in cells, and their normal function is necessary for ATP supply. A recent study has shown that mitochondrial damage and ATP depletion play a central role in the development of acute pancreatitis [10]. Therefore, we speculate that CBS is an important factor in maintaining mitochondrial function in AP.

Vitamin B_12_ (VB 12), also known as cobalamin, is an essential cofactor in humans that we obtain from our diet [11]. Failure of VB 12 supply leads to inadequate synthesis of S-Adenosylmethionine (SAM), an allosteric activator of CBS, which is difficult to maintain the activity of CBS [12, 13]. Existing research demonstrated that VB 12 administration significantly blunts the kidney damage caused by ischemia/reperfusion via marked suppression of ROS and associated inflammation and apoptotic cell death [14]. Quite apart from that, clinical studies have found that the administration of VB 12 has a better protective effect on atherosclerosis in patients lacking CBS [15]. However, the effect of VB 12 on CBS in acute pancreatitis is still unclear.

In the current study, the aims of this study were to determine (I) whether VB 12 attenuates the pathological damage of acute pancreatitis by CBS and (II) whether VB 12 inhibited oxidative stress and remedy mitochondrial dysfunction in acinar cells via CBS/SIRT1 signaling. We employed typical acute pancreatitis models *in vivo* and *in vitro* and found that VB 12 activated CBS-SIRT1 axis to suppress oxidative stress and repair mitochondrial damage in the pathological process of acute pancreatitis.

## 2. Materials and Methods

### 2.1. Reagents

Sodium taurocholate (NaT), hexadecyl trimethyl ammonium bromide (HETAB), and tetramethyl benzidine (TMB) were purchased from Sigma-Aldrich (St. Louis, USA). Hoechst 33342, propidium iodide (PI), and tetramethylrhodamine methyl ester (TMRM) were from Molecular Probes (Eugene, USA). Collagenase IV was purchased from Worthington Biochemical Corporation (Lakewood, USA). Protease inhibitor, ATP determination kit, RIPA, and 5-chloromethyl-2, 7-dichlorodihydrofluorescein diacetate acetyl ester (DCFH-DA) were purchased from Beyotime Biotech (Shanghai, China). Mouse TNF-*α* ELISA kit and mouse IL-1*β* ELISA kit were purchased from Elabscience Biotechnology Co. Ltd. (Wuhan, China). VB 12 was provided by Xinyi Jiufu Pharmaceutical Co., Ltd. (Shanghai, China). EX-527 was from Topscience Co., Ltd. (Shanghai, China).

### 2.2. Animals and Treatments

All studies involving animals are carried out in strict accordance with the Guide of Laboratory Animal Care and Use (Institute of Laboratory Animal Resources, 1996) and have been approved by the Ethics Committee of West China Hospital. Pathogen-free (SPF) male Balb/C mice (25-30 g; 6-8 weeks) were from Chengdu Dashuo Experimental Animal Co., Ltd. (Chengdu, China). 24 mice were randomly divided into four groups: control group (Con), acute pancreatitis group (NaT 3%, retrograde injection of 3% NaT into pancreaticobiliary duct), NaT + VB 12 (20 *μ*g/kg) group, and NaT +VB 12 (40 *μ*g/kg) group. Two doses of VB 12 were injected intraperitoneally at 1 hour, 3 hours, and 6 hours after modeling. The control and acute pancreatitis group were injected with the same volume of saline. Mice were killed 24 hours after operation, and serum and pancreatic tissue were collected.

### 2.3. Histopathological Analysis

According to the method of Shen et al. [16], the pathological changes of pancreatic tissue were analyzed. Fresh pancreatic tissue was fixed in 4% paraformaldehyde for 48 hours. The tissues were embedded in paraffin, cut into 5 *μ*m sections, and stained with hematoxylin and eosin (H&E). Each slide was observed under an optical microscope, and the pathological changes of pancreatic tissue were evaluated at 200 magnification. Two pathologists blindly evaluated the histopathological score of pancreas, and the scores of edema, inflammatory cell infiltration, and necrosis ranged from 0 to 3 [17].

### 2.4. Preparation of Pancreatic Acinar Cell

Primary acinar cells were isolated from adult male mice according to the method of Shen et al. [16]. Male Balb/c mice were dislocated and killed, and the pancreas was isolated and digested with collagenase IV (200 U/mL) at 37°C for 19 minutes. After incubation with collagenase IV, cells were separated by mechanical destruction of tissues, filtered through a 100 *μ*m cell filter, and then, centrifuged at 700 rpm for 2 minutes to obtain cell precipitates. Then, the cells were resuspended in an extracellular solution containing 140 mM NaCl, 4.7 mM KCl, 1.13 mM MgCl_2_, 1 mM CaCl_2_, 10 mM D-glucose, and 10 mM HEPES (adjusted to pH 7.35 with NaOH). Cells were treated at room temperature and used within 4 hours after separation.

### 2.5. Detection of Serum Amylase and Lipase

Blood samples were collected and centrifuged at 3000 rpm for 10 minutes, and 20 *μ*L serum was diluted to 200 *μ*L. Serum lipase and amylase were measured by automatic biochemical analyzer (Roche, Mannheim, Germany) according to manufacturer's instructions.

### 2.6. Detection of Inflammatory Factors

Enzyme-linked immunosorbent assay (ELISA) was used to detect the TNF-*α* and IL-1*β* in cell culture supernatant of mice. According to manufacturer's instructions, TNF-*α* and IL-1*β* were measured with commercial ELISA kits (Elabscience Biotechnology Co. Ltd., Wuhan, China), respectively. SpectraMax M5 microplate reader (Molecular Devices, LLC, Sunnyvale, CA, USA) was used to record the absorbance at 450 nm.

### 2.7. Detection of Necrotic Cell Death

The method has been described previously [18]. Fresh pancreatic acinar cells were treated with NaT (final concentration 5 mM) and incubated at room temperature for 50 minutes with or without various concentrations of VB 12 (60 nM, 100 nM). Then, acinar cells were treated with Hoechst 33342 (50 *μ*g/mL) and propidium iodide (PI; 1 *μ*mol/mL) to stain the total nucleus and the necrotic cells characterized by ruptured plasma membrane, respectively [19]. Automatic ZEISS AX10 imager A2/AX10 cam HRC (Jena GmbH, Germany) was used to record the images. The total number of acinar cells showing PI uptake was calculated from each condition, with a minimum of 1000 cells counted, to provide the percentage (necrosis %) with five isolates per condition.

### 2.8. Detection of GSH/GSSG

The ratio of GSH/GSSG in pancreatic tissue was detected by a commercial biochemical Kit (Jiancheng, Nanjing, China). After constructing AP model *in vivo* for 24 hours, the pancreatic tissue was collected and lysed into homogenate. The acinar cells in vitro were cultured for 50 min with or without VB 12 (60 nM, 100 nM), and the cells were collected and lysed into homogenate. The GSH/GSSG ratio is determined according to the reagent instructions.

### 2.9. Detection of Intracellular Reactive Oxygen Species

As previously mentioned [20], to measure the formation of ROS in acinar cells, the fluorescent probe DCFH-DA (10 *μ*M, excitation, 488 nm; emission, 505-550 nm) was used. Fresh acinar cells were loaded with DCFH-DA at 37°C in the dark for 20 minutes, washed twice with buffer solution and treated with NaT and VB 12 for the specified time. The processed cells were fixed on the slides, and the images were captured quickly with an automatic microscope. At least 10 fields were obtained, and the fluorescence intensity was analyzed by image J.

### 2.10. Detection of Mitochondrial Membrane Potential

According to manufacturer's instructions, tetramethylrhodamine methyl ester (TMRM,1 *μ*M) fluorescent probe is used to evaluate mitochondrial membrane potential. Acinar cells were incubated with NaT and VB 12 for a specified time and then loaded with TMRM and incubated at 37°C for 20 minutes in the dark. After loading, acinar cells were washed with buffer twice. The acinar cells were quickly placed under an automatic fluorescence microscope and measured at 543 nm (excitation) and 570 nm (emission). Finally, the fluorescence area was quantified by image J.

### 2.11. Measurement of ATP Level

The method has been described previously [18]. The ATP levels were measured by luminescent ATP detection assay system. In brief, cells were incubated with or without NaT (5 mM) and VB 12 (60 nM or 100 nM) for 30 minutes. After the treatment, acinar cells were resuspended in 200 *μ*L lysis buffer, boiled for 2 minutes, and then, centrifuged at 12 000 ×g at 4°C for 5 minutes. Luminescence in supernatant was detected using Synergy Mx multifunctional microplate reader (Gene Company Ltd., China). The data of each group were homogenized with protein concentration and then normalized to the control (100%).

### 2.12. Determination of Myeloperoxidase Activity

The method has been described previously [16]. Pancreatic tissue samples were homogenized (60 mg per mL) in 100 mM phosphate buffer (pH 7.4), centrifuged at 16 000 ×g for 15 minutes, and then, the precipitate was resuspended in 100 mM PBS (pH 5.4). The suspension was repeatedly freeze-thawed and ultrasonically treated. After centrifugation, supernatant samples (20 *μ*L containing 40 *μ*g protein) was incubated in a reaction solution consisting of 100 mM sodium phosphate buffer, 0.5% HETAB, and 2 mM TMB. The mixture was incubated at room temperature for 3 minutes and then hydrogen peroxide (0.01%; 50 *μ*L). The absorbance was measured at 655 nm with Synergy Mx multifunctional microplate reader, and MPO activity was calculated as the difference between absorbance at 0 and 3 minutes. The data were normalized to protein concentration, and then, 100% of each model was normalized to AP group.

### 2.13. Western Blot Analysis

The method has been described previously [18]. Protein lysate from pancreatic tissue or isolated pancreatic acinar cells was prepared by homogenizing in RIPA containing protease and phosphatase inhibitor. Lysate samples (20 *μ*g protein) were loaded on 15% polyacrylamide gel, then the gel was transferred to polyvinylidene fluoride (PVDF) membrane, and finally, blocked and incubated with primary antibody and secondary antibody. The images were detected by enhanced chemiluminescence detection system (California, USA). Anti-CBS (1 : 300) and anti-Nrf2 (1 : 1000) were purchased from Bioss (Beijing, China); anti-SIRT1 (1 : 1000) and anti-PGC1-*α* (1 : 1000) were purchased from Wanleibio (Shenyang, China); anti-phospho-P38 (1 : 1000), anti-P38 (1 : 1000), anti-LC3 (1 : 1000), anti-*β*-actin (1 : 1000), and anti-P62 (1 : 1000) were purchased from Cell Signaling Technology (Danvers, USA); anti-Parkin (1 : 1000) was from Santa Cruz Biotechnology (California, USA).

### 2.14. Statistical Analysis

Values are presented as the mean ± SEM. Statistical analysis was performed using GraphPad Prism 5.0 (GraphPad Software, San Diego, CA). Comparisons between two groups were performed by Student's *t*-tests. The results were calculated using data from three independent experiments. *P* < 0.05 was considered statistically significant.

## 3. Results

### 3.1. VB 12 Improves Pancreatic Damage in Acute Pancreatitis by Activating CBS

To explore the role of CBS in acute pancreatitis, we constructed a classic AP model induced by sodium taurocholate (NaT). We found that NaT treatment caused pancreatic enlargement and whitish lesions (Fig. S10A) and a sharp increase in serum pancreatic amylase and lipase (Fig. S10B). The results of H&E staining and tissue scoring showed obvious tissue edema, inflammatory infiltration, and necrosis in the AP group (Fig. S10C and D). What is more, we detected the decrease of CBS expression in pancreatic tissue (Fig. S10E). Consistent with previous studies, NaT treatment also reduced the ratio of GSH/GSSG (Fig. S10H), an important indicator for maintaining cell's redox environment. These results demonstrate that CBS is closely related to pancreatic damage in acute pancreatitis.

**Figure 1:**
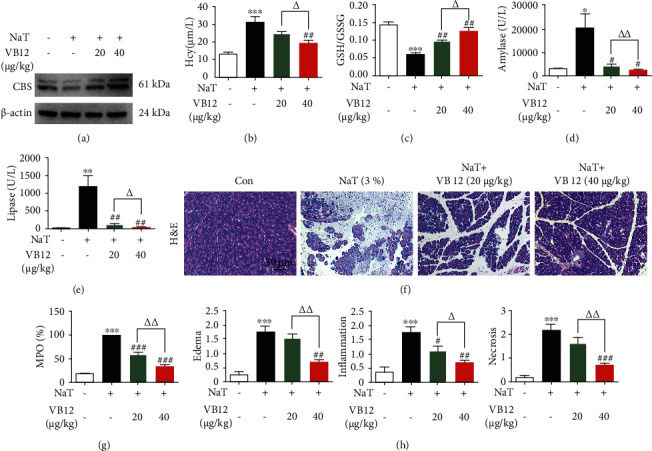
VB 12 improves pancreatic damage in acute pancreatitis by activating CBS. Animals were randomly divided into four experimental groups: AP group treated with NaT (3%), NaT + VB 12 (20 *μ*g/kg, ip), NaT + VB 12 (40 *μ*g/kg, ip) and control group treated with 0.9% saline (*n* = 6). NaT (3%) was injected through the pancreaticobiliary tract, and pancreatic tissue and blood were collected at 24 hours for follow-up studies. (a) The expression of CBS in pancreatic tissue was measured by Western blot. (b) The level of Hcy in serum was detected by ELISA assay kit. (c) The GSH/GSSG ratio in pancreatic tissue was detected by assay kit. (d, e, g) The levels of serum amylase, lipase, and pancreatic myeloperoxidase activity were detected. (f) Representative histopathological sections of pancreatic tissues by hematoxylin-eosin staining. Scale bar: 50 *μ*m. (h) The histological scores of pancreatic edema, inflammatory infiltration, and necrosis were obtained to evaluate the degree of injury. Slides were evaluated by two pathologists in a blinded manner. The results are presented as the mean ± SEM. ^∗^*P* < 0.05, ^∗∗^*P* < 0.01, ^∗∗∗^*P* < 0.001 vs. Con group. ^#^*P* < 0.05, ^##^*P* < 0.01, ^###^*P* < 0.001 vs. NaT group. ^∆^*P* < 0.05, ^∆∆^*P* < 0.01, ^∆∆∆^*P* < 0.001 vs. VB 12(20 *μ*g/kg) treatment group. Con: control group; NaT: acute pancreatitis group.

In view of the protection of VB 12 in CBS deficiency disease [2], we exogenously administered VB 12 into mice to evaluate whether it is beneficial to AP. Firstly, we detected that the expression of CBS and GSH/GSSG ratio in pancreatic tissue were significantly increased, while the level of Hcy was decreased (Figures 1(a)–1(c)). Simultaneously, we found that VB 12 decreased serum amylase and lipase levels, and inhibited MPO activity in a dose-dependent manner (Figures 1(d), 1(e), and 1(g)). VB 12 also significantly improved the total histology score and reduced the degrees of edema, inflammation, and necrosis (Figures 1(f) and 1(h)). Based on the above results, we could conclude that CBS is a potential target for VB 12 to protect NaT-induced acute pancreatitis.

### 3.2. VB 12 Reduces Necrosis and Inflammatory Response of Pancreatic Acinar Cells by Activating CBS

**Figure 2:**
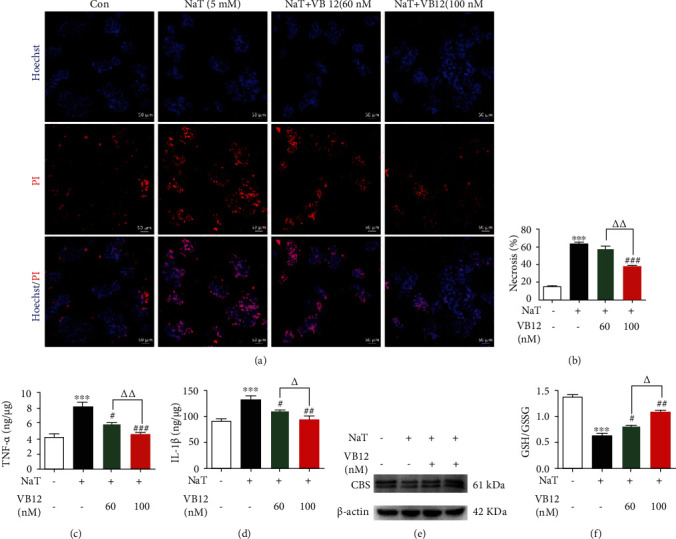
VB 12 reduces necrosis and inflammatory response of pancreatic acinar cells by activating CBS. (a) Representative images showing Hoechst 33342 (blue) and PI (red) staining in pancreatic acinar cells stimulated with NaT (5 mM) in the absence or presence of VB 12 (60,100 nM). (b) The necrosis rate was quantified by image J. (c, d) The levels of TNF-*α* and IL-1*β* in the lysate of acinar cells were detected by ELISA kit. (e) The expression of CBS protein in acinar cells was detected by Western blot. (f) The GSH/GSSG ratio in acinar cells was detected by assay kit. Data are expressed as means ± SEM, experiments were repeated more than three times. ^∗∗∗^*P* < 0.0001 vs. Con group, ^##^*P* < 0.01 vs. NaT group, ^###^*P* < 0.0001 vs. NaT group, ^∆∆^*P* < 0.01 vs. VB 12 (60 nM) group, ^∆∆∆^*P* < 0.0001 vs. VB 12 (60 nM) group. Con: control group; NaT: acute pancreatitis group.

Considering that necrosis is the main way of acinar cell death [21], we evaluated the effect of VB 12 on necrosis of primary acinar cells. The experimental results showed that NaT caused a large number of necrosis of acinar cells, while VB 12 (100 nM) significantly reduced the necrosis rate of acinar cells (Figures 2(a) and 2(b)). The massive production of cytokines such as TNF-*α* leads to necrosis of acinar cells and accelerates the occurrence of acute pancreatitis [22–24]. As shown in Figures 2(c) and 2(d), the production of TNF-*α* and IL-1*β* in cell culture supernatant of mice was significantly increased by NaT treatment, which were inhibited by VB 12 in a dose-dependent manner. In addition, compared with AP group, VB 12 significantly increased the expression of CBS (Figure 2(e)) and the ratio of GSH/GSSG (Figure 2(f)), which was consistent with the experimental results in vivo. The experiments reveal that the necrosis and inflammatory response of pancreatic acinar cells related to CBS deletion was significantly reduced by VB 12.

### 3.3. VB 12 Inhibits Oxidative Stress in Acinar Cells by Activating CBS and SIRT1

**Figure 3:**
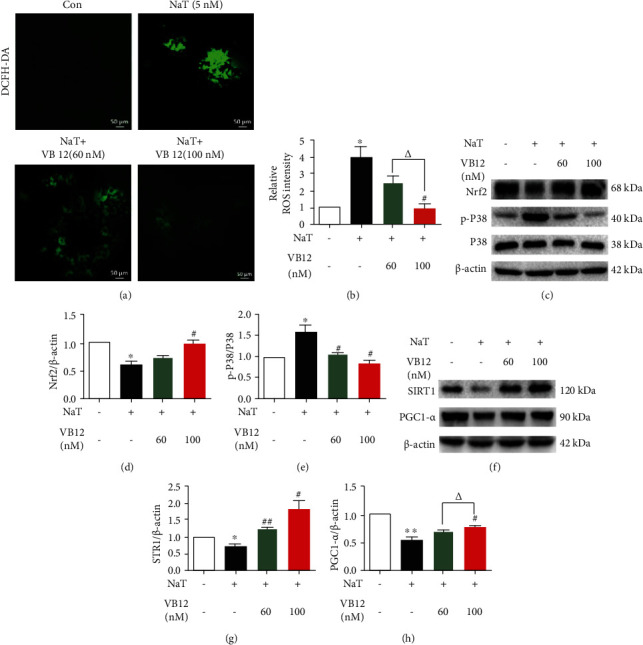
VB 12 inhibits oxidative stress in acinar cells by activating CBS and SIRT1. (a, b) Fresh isolated acinar cells were incubated with DCFH-DA (1 *μ*M) in dark for 20 minutes, washed twice, and then, incubated with NaT (5 mM) alone or combined with VB 12 (60 nM, 100 nM) for 30 minutes. Fluorescence microscopy was used to capture the fluorescence intensity of ROS in cells and quantify the level of total ROS in cells. (c–e) Western blot analysis of protein levels of p-p38, p38, and Nrf2 in acinar cells treated with NaT, NaT + VB 12 (60 nM), and NaT + VB 12 (100 nM). (f–h) The expression of SIRT1 and PGC1-*α* protein in acinar cell lysate were analyzed. Data are expressed as means ± SEM; experiments were repeated more than three times. ^∗^*P* < 0.05 vs. Con group, ^∗∗^*P* < 0.01 vs. Con group, ^#^*P* < 0.05 vs. NaT group,^##^*P* < 0.01 vs. NaT group, ^∆^*P* < 0.05 vs. VB 12 (60 nM) group, ^∆∆^*P* < 0.01 vs. VB 12 (60 nM) group. Con: control group; NaT: acute pancreatitis group.

CBS is the rate-limiting enzyme in the synthesis of GSH (an important antioxidant), and its downregulation promotes inflammation and oxidative stress. Given that the accumulation of reactive oxygen species is a sign of oxidative stress, we detected whether VB 12 could inhibit the oxidative stress of acinar cells by activating CBS. As expected, VB 12 suppressed the increase in intracellular ROS caused by NaT treatment in a dose-dependent manner (Figures 3(a) and 3(b)). Various transcription factors in the antioxidant system are regulated by Nrf2 to maintain the redox homeostasis of cells and protect cells from oxidative damage. Western blot analysis showed that VB 12 inhibited the decrease of Nrf2 expression in acinar cells exposed to NaT (Figures 3(c) and 3(d)). Similarly, p-P38, a marker of oxidative stress, increased in the NaT treatment group, while VB 12 inhibited its expression (Figures 3(c) and 3(e)). These results indicate that the occurrence of oxidative stress in acute pancreatitis may be due to the lack of CBS and could be improved by VB 12.

SIRT1-mediated mitochondria dysfunction closely related to failure of ATP and ROS homeostasis [25]. Therefore, we further explore whether SIRT1 is involved in oxidative stress in AP. The results showed that NaT treatment caused the downregulation of SIRT1 as well as its direct downstream target PGC1-*α* in acinar cells, and both were increased by VB 12 in a concentration-dependent manner (Figures 3(f)–3(h)). These results support that SIRT1 plays a role in the treatment of VB 12.

### 3.4. VB 12 Regulates SIRT1 to Inhibit Oxidative Stress by Activating CBS

**Figure 4:**
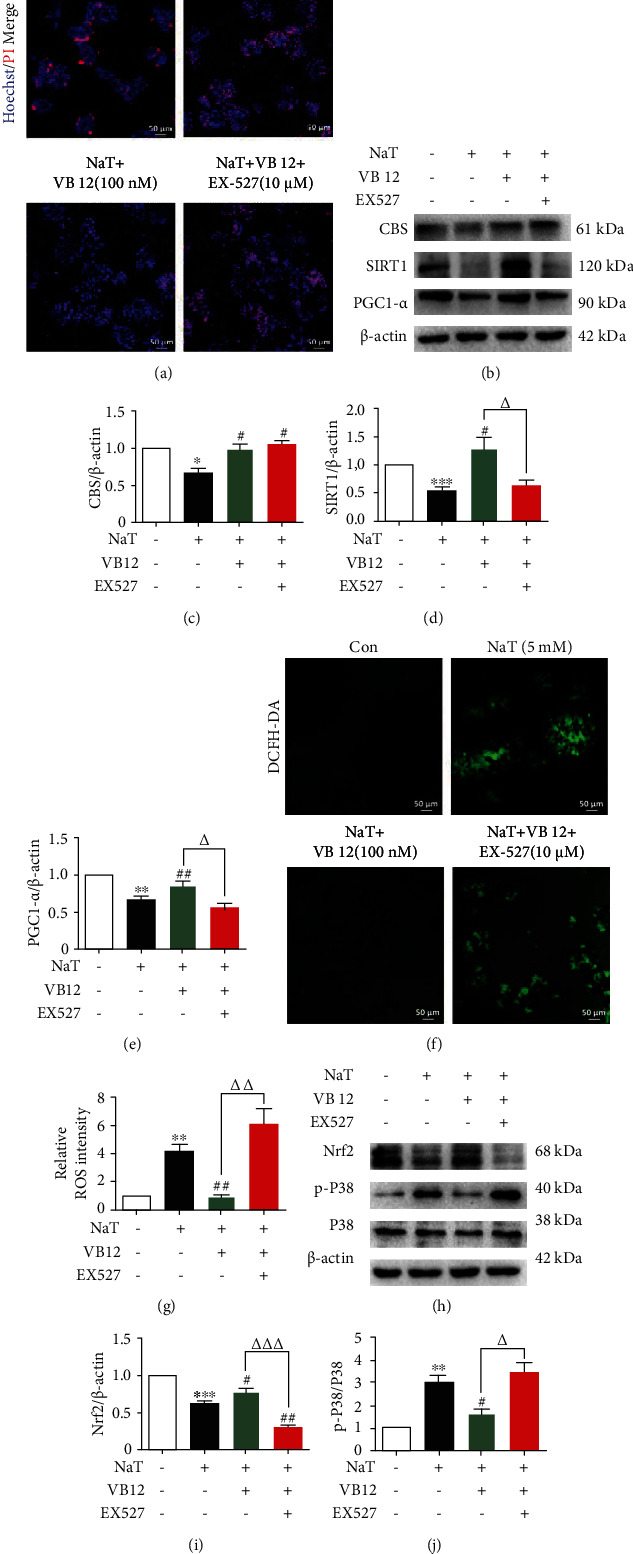
VB12 regulates SIRT1 to inhibit oxidative stress by activating CBS. (a) Representative images showing Hoechst 33342 (blue) and PI (red) staining in pancreatic acinar cells treated with NaT, NaT+VB 12, or NaT+VB 12+EX-527 for 50 minutes, respectively. (b) The acinar cells were treated with NaT, NaT+VB 12, or NaT+VB 12+EX-527, respectively, and the protein levels of CBS, SIRT1, and PGC1-*α* were determined by Western blot analysis. (c–e) Relative band densities were quantified, and the normalized values are indicated in the histogram. (f, g) Representative images of ROS staining in acinar cells with DCFH-DA. (h–j) The protein expression changes of p-P38, P38, and Nrf2 in acinar cells treated with EX-527 were analyzed and quantified. ^∗^*P* < 0.01 vs. Con group, ^∗∗∗^*P* < 0.0001 vs. Con group, ^∗∗^*P* < 0.01 vs. Con group, ^##^*P* < 0.01 vs. NaT group, ^#^*P* < 0.05 vs. NaT group, ^∆^*P* < 0.05 vs. VB 12 (100 nM) group, ^∆∆^*P* < 0.01 vs. VB 12 (100 nM) group, ^∆∆∆^*P* < 0.0001 vs. VB 12 (100 nM) group. Con: control group; NaT: acute pancreatitis group. All the experiments were repeated three times with similar results.

Previous studies have shown that the expression of SIRT1 is downregulated in CBS knockout cells [8], but the relationship between the two is unclear. Therefore, we explored whether CBS is involved in the regulation of SIRT1 in acinar cells. The results of PI/Hoechst staining showed that the reduced acinar cell necrosis of VB 12 was offset by an effective and specific SIRT1 inhibitor EX-527 (Figure 4(a)). Furthermore, we found that loaded EX-527 inhibited the expression of SIRT1 and its downstream target PGC1-*α*, but did not disturb the effect of VB 12 on CBS (Figures 4(b)–4(e)), which proves that SIRT1 is downstream of the CBS signaling pathway. In addition, the protective effect of VB 12 on oxidative stress is also weakened with the addition of EX-527, which is mainly manifested by increased ROS levels (Figures 4(f) and 4(g)) and decreased expression of oxidative stress-related protein Nrf2, companied with the phosphorylation of P38 increased (Figures 4(h)–4(j)). These data indicate that VB 12 inhibits oxidative stress in acinar cells by activating CBS and its downstream target SIRT1.

### 3.5. VB 12 Repairs Mitochondrial Dysfunction Related to Oxidative Stress by Activating the CBS/SIRT1 Axis

**Figure 5:**
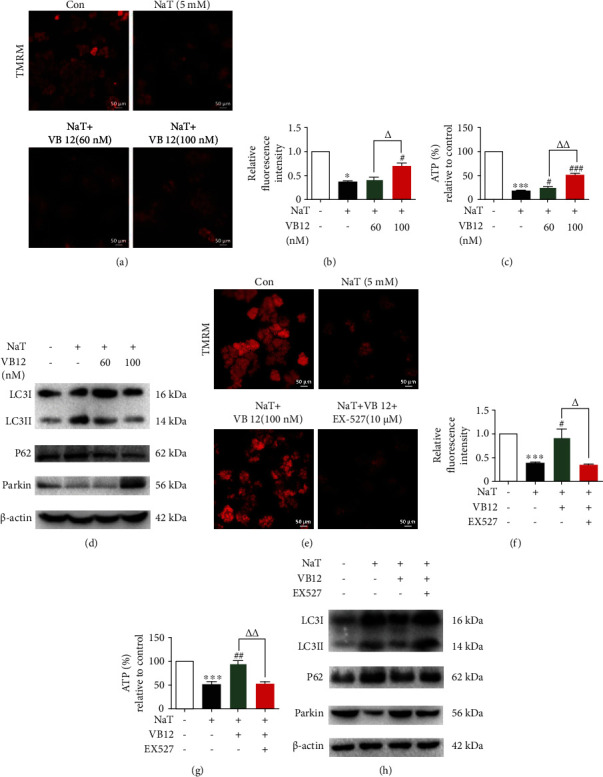
VB 12 repairs mitochondrial function by activating the CBS/SIRT1 axis. (a, b) Representative image of ΔΨm in acinar cells stained with TMRM and quantification of the fluorescence density of ΔΨm. (c) ATP levels were measured by luminescence in pancreatic acinar cells; data were normalized to untreated control as 100%. (d) Western blot analysis showed the effect of VB 12 on SQSTM1(p62), LC3II, and Parkin protein levels in acinar cells. After incubating the cells with EX-527, the TMRM staining (e, f), ATP level (g), and the expression of SQSTM1 (p62), LC3II/LC3I, and Parkin protein (h) in each group were detected. ^∗^*P* < 0.05 vs. Con group, ^∗∗∗^*P* < 0.0001 vs. Con group, ^#^*P* < 0.05 vs. NaT group, ^∆^*P* < 0.05 vs. VB 12 (100 nM) group, ^∆∆^*P* < 0.01 vs. VB 12 (100 nM) group. Con: control group; NaT: acute pancreatitis group. All the experiments were repeated three times with similar results.

The silencing of CBS-mediated mitochondrial dysfunction in endothelial cells has been studied, and vitamin B_12_ is necessary for the tricarboxylic acid cycle [26], that is, to maintain the function of mitochondria in cells. Therefore, we presume that VB 12 could improve mitochondrial impairment related to CBS deficiency in acinar cells. NaT treatment caused the loss of membrane potential (ΔΨm) (Figures 5(a) and 5(b)) and the decrease of ATP level (Figure 5(c)) in acinar cells, which could be reversed by VB 12 in a dose-dependent manner. Mitophagy is defined as the selective elimination of damaged mitochondria or excess mitochondria to maintain the integrity of the mitochondrial network and promote cell homeostasis and survival [27]. Parkin, the main regulator of mitochondrial autophagy, was significantly reduced, and the expression of autophagy-related protein LC3II and autophagy adaptor protein P62 was increased after NaT loading, while VB 12 treatment increased the expression of Parkin and decreases the levels of LC3II and P62 (Figure 5(d)). These results confirm our conjecture that VB 12 may repair mitochondrial function by promoting mitophagy. SIRT1 is not only involved in the regulation of oxidative stress but also as an energy sensor in the regulation of mitochondrial function [28]. EX-527 treatment inhibited the effect of VB 12 on the improvement of ΔΨm (Figures 5(e) and 5(f)) and ATP (Figure 5(g)) and downregulated the expression of parkin and increased the expression of LC3II and P62 (Figure 5(h)), similar to NaT treatment. Therefore, we could conclude that mitochondrial dysfunction related to CBS deficiency is alleviated by VB 12 involving SIRT1 activation.

### 3.6. VB 12 Also Activates the SIRT1 Pathway In Vivo

**Figure 6:**
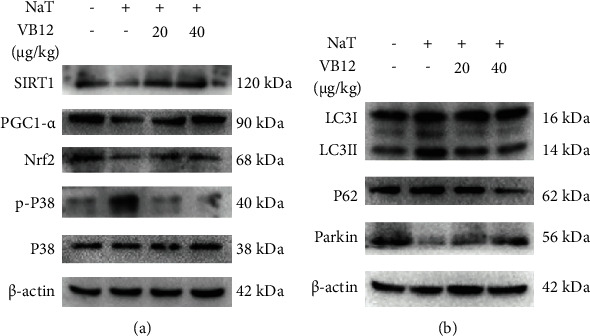
VB 12 also activates the SIRT1 pathway *in vivo* of acute pancreatitis mice. Animals were randomly divided into four experimental groups: AP group treated with NaT (3%), NaT + VB 12 (20 *μ*g/kg, ip), NaT + VB 12 (40 *μ*g/kg, ip), and control group treated with 0.9% saline (*n* = 6). NaT (3%) was injected through the pancreaticobiliary tract, and pancreatic tissue and blood were collected at 24 hours for follow-up studies. (a, b) The expression of SIRT1, PGC1-*α*, Nrf2, p-P38, P38, and mitophagy-related proteins in pancreatic tissue was measured by Western blot. Con: control group; NaT: acute pancreatitis group. All the experiments were repeated three times with similar results.

Finally, we further verified the important role of SIRT1 signal axis in acute pancreatitis. As shown in Figure 6, NaT treatment caused downregulation of SIRT1/PGC1*α*, Nrf2, and Parkin protein expression, and upregulation of p-P38/P38, LC3II/LC3I, and P62 protein expression in pancreatic tissue, while VB 12 reversed these changes (protein quantification results in Fig. S9). These results indicate that VB 12 does protect pancreatic tissue from NaT damage through the CBS/SIRT1 signal axis.

## 4. Discussion

Acute pancreatitis is an inflammatory process that could cause distal organ failure and leads to a higher mortality rate. Therefore, it has long been a major concern for clinical gastroenterologists. In our study, we first confirmed that the key regulatory protein CBS of oxidative stress and the ratio of GSH/GSSG, a reliable indicator of oxidative stress, were downregulated in AP models *in vivo* and *in vitro*. The pancreatic damage and acinar cell necrosis related to CBS deficiency are significantly improved by VB 12, which stimulates ROS scavenging by preserving GSH. Therefore, CBS is a potential target of VB 12 in the treatment of acute pancreatitis. EX-527 exposure counteracted the protective effects of VB 12 by promoting oxidative stress and mitochondrial damage, further illustrating the positive regulation of SIRT1 by CBS.

CBS regulates homocysteine metabolism and contributes to GSH biosynthesis through which it plays multifunctional roles in the regulation of cellular energetics, redox status, DNA methylation, and protein modification [29]. Clinically, patients with untreated CBS deficiency suffer from various diseases, including hyperhomocysteinemia [30], thrombosis, osteoporosis, and mental retardation [31, 32]. In this study, we observed the downregulation of CBS expression, Hcy accumulation, and pancreatic injury in AP model. At the same time, we also found that the level of serum H_2_S in AP model was significantly decreased (Fig. S11), which was consistent with the previous studies [33]. After intervention with VB 12, the expression of CBS and the level of H_2_S were significantly increased, the accumulation of Hcy was reduced, and the pancreatic tissue was well protected. This is consistent with previous studies that VB 12 has a significant effect on atherosclerosis in patients with CBS deficiency [15]. Since that acute pancreatitis is an inflammatory disorder of the pancreas, we have also detected that VB 12 reduced the level of MPO in the AP model in vivo and reduced the level of inflammatory factors IL-1*β* and TNF-*α* in the supernatant of acinar cell culture *in vitro*. Our results indicate that pancreatic damage related to CBS deficiency could be better alleviated by VB 12.

Oxidative stress, a common biological response for damage, contributes to the severity of AP [34]. Reduced glutathione (GSH) is the major nonprotein thiol in mammalian cells and plays a central role as antioxidant. It is in equilibrium with oxidized glutathione (GSSG), and the ratio between GSSG and GSH is a reliable indicator of oxidative stress because it reflects the balance between antioxidant status and prooxidant reactions in cells. CBS has been found to modulate redox homeostasis, and the knockdown of CBS gene promotes inflammation and oxidative stress. Thus, we suspect that VB 12 may combat oxidative stress by increasing the CBS in AP, which is confirmed by the change of reactive oxygen species and the ratio GSH/GSSG. Considering that oxidative stress is characterized by abnormal expression of major regulatory genes, we detected that Nrf2 expression decreased, and phosphorylated P38 increased in AP, and the application of VB 12 make a reversion. The effect of VB 12 on ROS levels and oxidative stress-related proteins indicates that CBS plays an important role in regulating oxidative stress in AP.

SIRT1 is a NAD^+^-dependent protein deacetylase distributed in cytoplasm and nucleus, regulating cellular oxidative stress burden and its toxicity [25, 35]. It has been reported that H_2_S inhibits oxidative stress by activating SIRT1 signal transduction to protect airway from cigarette smoke damage in mice [36]. Therefore, we suppose that SIRT1 signal may be involved in the regulation of oxidative stress by CBS in acinar cells. Here, downregulated SIRT1 and its downstream direct target PGC1-*α* were found in acinar cells, and our findings verified that SIRT1 is a downstream target of CBS in acinar cells induced by NaT. Although SIRT1 is mainly a nuclear protein, SIRT1 deacetylation of peroxisome proliferator-activated receptor PGC-1*α* has been widely involved in metabolic control and mitochondrial biogenesis, which was considered as part of the basis of SIRT1 in energy limitation. In line with this, our findings indicate that NaT exposure induced mitochondrial dysfunction in acinar cells characterized by decreased ATP synthesis and loss of mitochondrial membrane potential. Since VB 12 is a necessary coenzyme factor for the mitochondria to carry out the tricarboxylic acid cycle, Ghemrawi et al. also found that the reduced cell utilization of VB 12 led to the abnormal response of organelles mediated by the decreased expression of SIRT1 [37]. We speculate that SIRT1 may be involved in the protective effect of VB 12 on mitochondria in acinar cells. Consistent with our expectation, EX-527, an inhibitor of SIRT1, eliminated the protective effect of VB 12 on mitochondria. Similarly, we also validated the SIRT1 pathway in pancreatic tissues in vivo. Our results are consistent with *in vitro* results. VB12 could regulate oxidative stress-related proteins and improve mitochondrial autophagy by increasing the expression of SIRT1. As far as we know, this is the first time that SIRT1 expression is closed related to VB 12 in AP.

As metabolic hubs, mitochondria facilitate crosstalk between the metabolic state of the cell by regulating autophagy [38]. Mitophagy, that is, an autophagic response that specifically targets mitochondria, is arguably the best characterized type of selective autophagy and an important way to maintain mitochondrial function [39, 40]. The expression of Parkin, a marker protein of mitochondrial autophagy, was significantly decreased in CBS-deleted acinar cells. According to reports, Parkin ubiquitinated several proteins and then recognized by ubiquitin binding proteins, which recruited mitochondria into autophagy pathway [41]. In accordance with this, the lipidation level of LC3, a key protein that contributes to the main steps of autophagy, and the expression of autophagy adaptor protein P62 increased, which is also dispensable for parkin-mediated mitophagy. Interestingly, VB 12 significantly promoted mitophagy in acinar cells. This may be due to the fact that VB 12 activates SIRT1 in acinar cells, while EX-527 directly inhibits the activation of Parkin, the lipidation effect of LC3, and the protein degradation reaction. In conclusion, these results strongly suggest the importance of SIRT1 in promoting mitophagy in CBS-deficient acinar cells.

In summary, this is the first study to demonstrate that VB 12 inhibits pancreatic injury caused by oxidative stress and mitochondrial dysfunction in a CBS-SIRT1-dependent manner. Our study provides basic evidence that targeting CBS/SIRT1 pathway might be a therapeutic strategy for acute pancreatitis.

## Data Availability

The data used to support the findings of this study are included within the article.

## Abbreviations

AP: Acute pancreatitis
CBS: Cysthionine-*β*-synthase
GSH: Glutathione
VB 12: Vitamin B_12_
ROS: Reactive oxygen species
SAM: S-Adenosylmethionine
H&E: Hematoxylin and eosin
NaT: Sodium taurocholate
MPO: Myeloperoxidase
PI: Propidium iodide
TMRM: Tetramethylrhodamine methyl ester
HETAB: Hexadecyl trimethyl ammonium bromide
TMB: Tetramethyl benzidine.

## Supplementary Materials

Supplementary Materials Fig. S1: illustrate the original Western blot bands. Fig. S2: original Western blot bands in Figures 2 and 3. Fig. S3: original Western blot bands in Figure 4. Fig. S4: original Western blot bands in Figure 5. Fig. S5: original Western blot bands in Figure 6. Fig. S6: VB 12 protect against NaT-induced activation of the necrotic cell death pathway in pancreatic acinar cells isolated from Balb/c mice. Fig. S7: serum VB12 concentration after intraperitoneal administration. Fig. S8: clarify the quantitative results of LC3II/LC3I ratio in Figure 5. Fig. S9: clarify the quantitative results of protein bands in Figure 6. Fig. S10: CBS loss is associated with pancreatic injury in AP mice. Fig. S11: VB 12 increases the content of H_2_S in serum.
