# Supplementary material for: Vitamin B12 Attenuates Acute Pancreatitis by Suppressing Oxidative Stress and Improving Mitochondria Dysfunction via CBS/SIRT1 Pathway
Source: Oxid Med Cell Longev. 2021 Dec 9;2021:7936316. doi: 10.1155/2021/7936316 (PMC8677375; doi:10.1155/2021/7936316)
Supplement: Supplementary Materials — Fig. S1: illustrate the original Western blot bands. Fig. S2: original Western blot bands in Figures 2 and 3. Fig. S3: original Western blot bands in Figure 4. Fig. S4: original Western blot bands in Figure 5. Fig. S5: original Western blot bands in Figure 6. Fig. S6: VB 12 protect against NaT-induced activation of the necrotic cell death pathway in pancreatic acinar cells isolated from Balb/c mice. Fig. S7: serum VB12 concentration after intraperitoneal administration. Fig. S8: clarify the quantitative results of LC3II/LC3I ratio in Figure 5. Fig. S9: clarify the quantitative results of protein bands in Figure 6. Fig. S10: CBS loss is associated with pancreatic injury in AP mice. Fig. S11: VB 12 increases the content of H2S in serum. [file 7936316.f1.doc]

**Vitamin B12 attenuates acute pancreatitis by suppressing oxidative stress and improving mitochondria dysfunction via CBS/SIRT1 pathway**

Jiyan Yuan^a,1^, Zeliang Wei ^a, b,1^, Guang Xin^a^, Xubao Liu^c^, Zongguang Zhou^d^, Yi Zhang^e^, Xiuxian Yu^a^, Chengyu Wan^a^, Qingqu Chen^a^, Weiyu Zhao^a^, Xueling Wang^f^, Yuman Dong^a^, Zhen Chen^a^, Xiaoting Chen^a^, Hai Niu^a^, Wen Huang^a*^

^a^ Laboratory of Ethnopharmacology, West China School of Medicine, West China Hospital, Sichuan University, Chengdu, Sichuan, China.

^b^ Department of Dermatology, West China Hospital, Sichuan University, Chengdu, Sichuan, China

^c^ Department of Pancreatic Surgery, West China Hospital of Sichuan University, Chengdu, Sichuan, China.

^d^ Department of Gastrointestinal Surgery and Laboratory of Digestive Surgery, West China Hospital, Sichuan University, Chengdu, China.

^e^ Research Core Facility of West China Hospital, Sichuan University, Chengdu Sichuan, China.

^f^ Integrated Chinese and Western Medicine Department, West China Hospital, Sichuan University, Chengdu, Sichuan, China.

*Corresponding author: Dr. Wen Huang

E-mail: huangwen@scu.edu.cn

Keyuan Road 4 No.1, Gaopeng Avenue, Gaoxin District, Chengdu, Sichuan, 610041, China.

Fax: +86-028-85164073, Tel: +86-028-85164076

^1^ These authors contributed equally to this work.

**Supplementary Information**


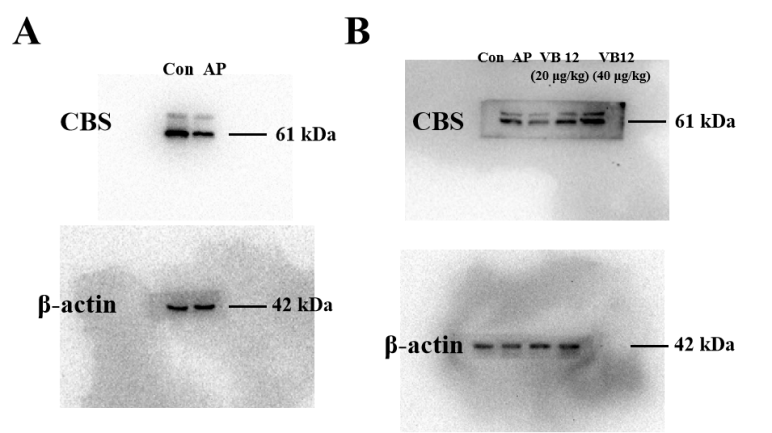


**Fig. S1.** **Illustrate the original western blot bands.** (A, B) All (ie, uncropped) western blot images of CBS and β-actin from Fig.S10 and Fig. 1, respectively. The experiment was repeated three times with similar results.


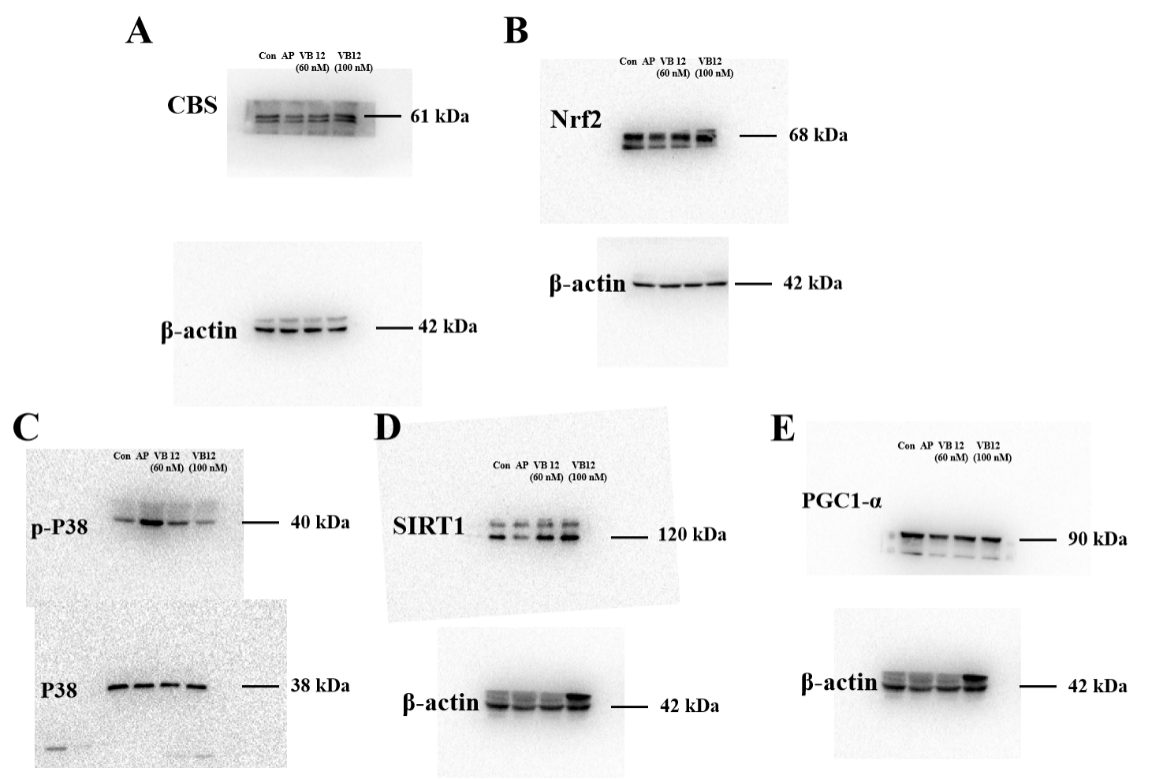


**Fig. S2. Original western blot bands in** **Fig.2 and Fig.3.** (A) Full (ie, uncropped) Western blot image from the CBS protein in Fig. 2. (B, C, D, E) All (ie, uncropped) Western blot images of Nrf-2, p-P38, P38, SIRT1, and PGC1-α proteins in Fig.3. The experiment was repeated three times with similar results.


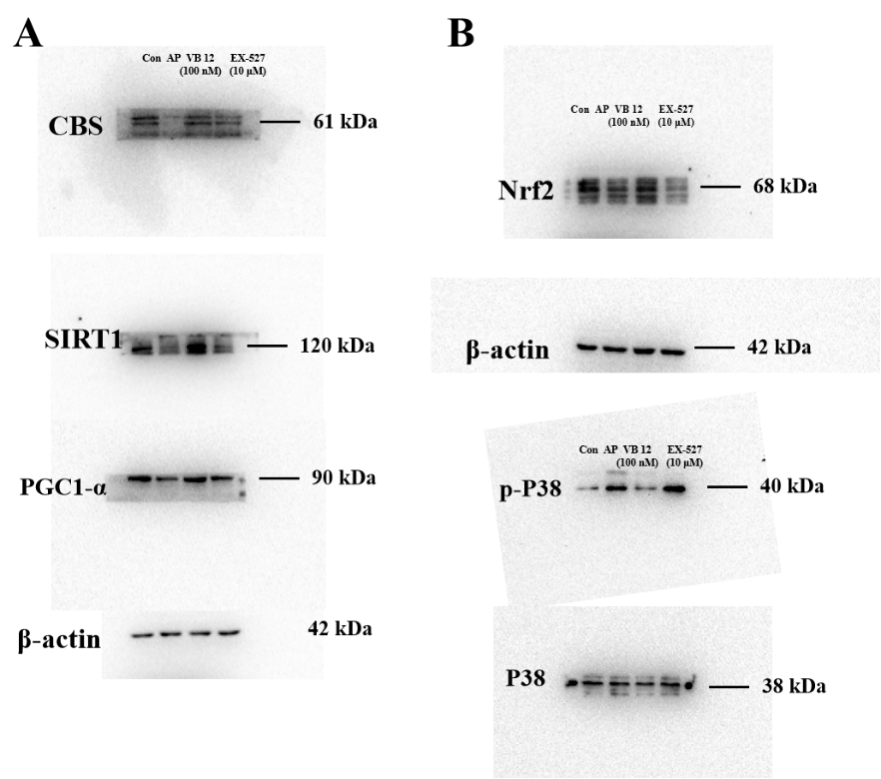


**Fig. S3. Original western blot bands in Fig.4.** (A, B) Western blot images of all (ie, uncropped) CBS, Nrf-2, p-P38, P38, SIRT1, and PGC1-α proteins in Fig. 4. The experiment was repeated three times with similar results.


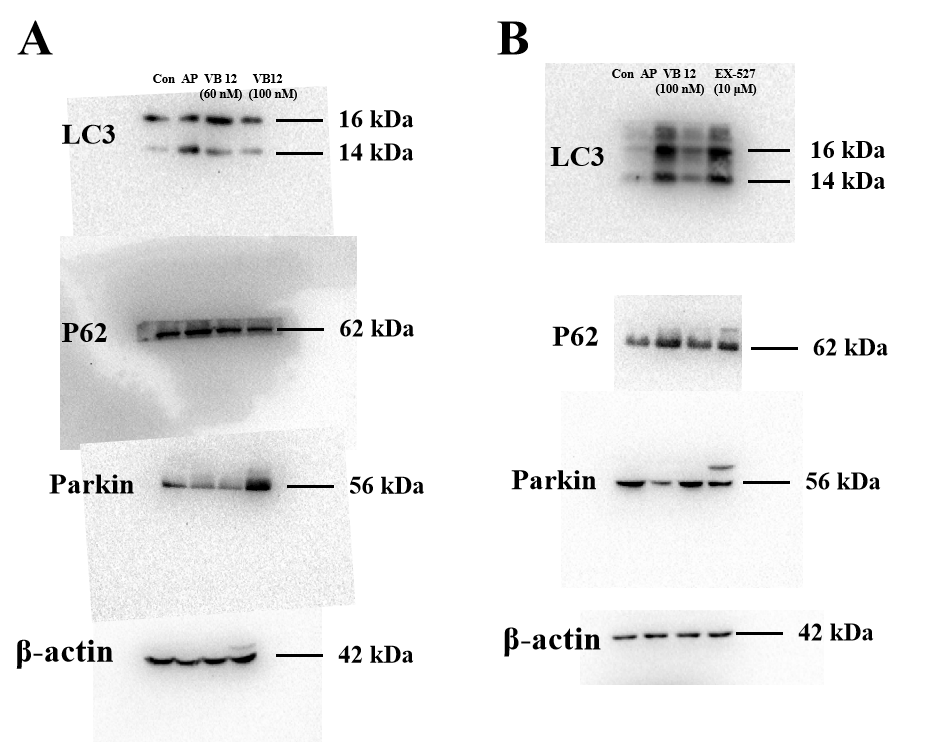


**Fig. S4. Original western blot bands in Fig.5.** (A) Western blot images of all (ie, uncropped) LC3, P62 and Parkin proteins in Fig. 5D. (B) Western blot images of all (ie, uncropped) LC3, P62 and Parkin proteins in Fig. 5H. The experiment was repeated three times with similar results.


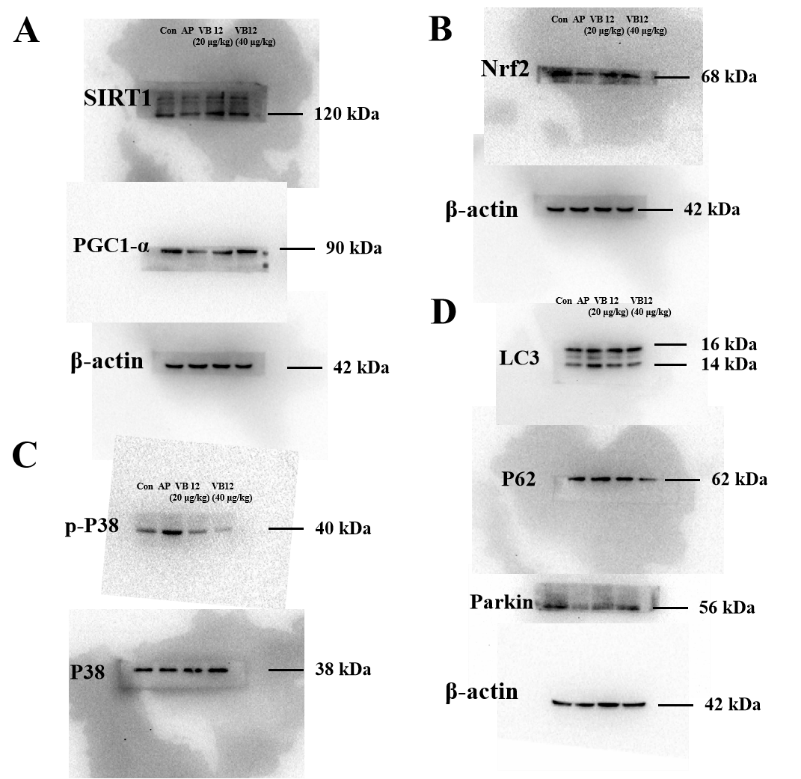
**Fig. S5. Original western blot bands in Fig.6.** (A, B, C, D) Western blot images of all (ie, uncropped) SIRT1, PGC1-α, Nrf-2, p-P38, P38, LC3, P62 and Parkin proteins in Fig. 6. The experiment was repeated three times with similar results.


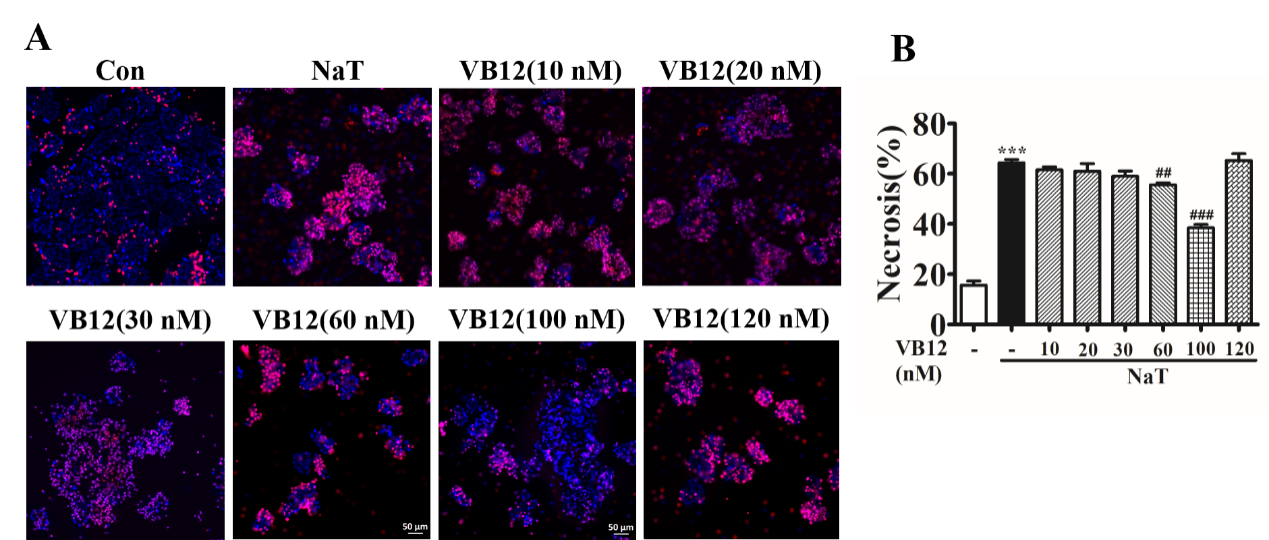


**Fig. S6.** **VB 12 protect against NaT-induced activation of the necrotic cell death pathway in pancreatic acinar cells isolated from Balb/c mice.** （A）Representative images showing Hoechst 33342 (blue) and PI (red) staining in pancreatic acinar cells stimulated with NaT (5 mM) in the absence or presence of VB 12 (10, 20, 30, 60,100, 120 nM). (B) The necrosis rate was quantified by image J. Data are expressed as means ± SEM, experiments were repeated more than three times. ****P* < 0.0001 vs. Con group, ^##^*P* < 0.01 vs. NaT group, ^###^*P* < 0.0001 vs. NaT group. Con: control group. NaT: acute pancreatitis group.

**Fig. S7.** **Serum VB12 concentration after intraperitoneal administration**
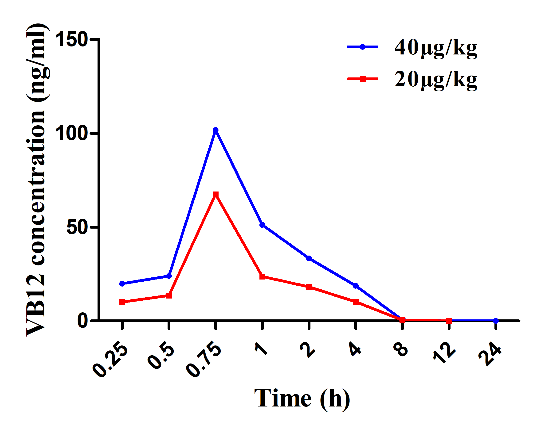
**.**


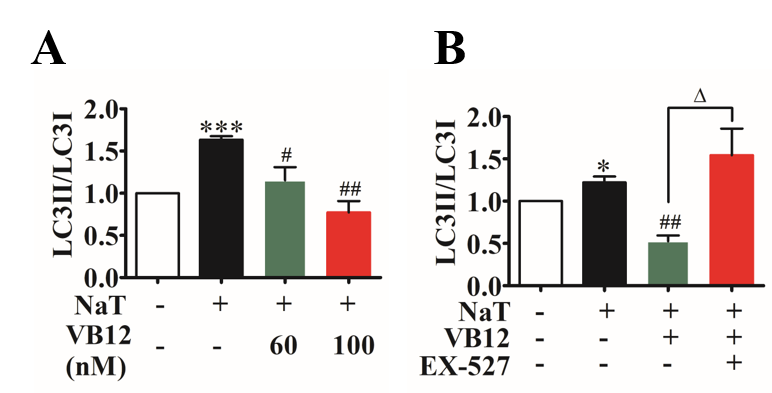


**Fig. S8. Clarify the quantitative results of LC3II/LC3I ratio in Fig. 5.** (A-B) Densitometry analysis results of LC3II/LC3I proteins. Presented values are means ± SEM. **P*< 0.05 vs. Con group, ****P*< 0.0001 vs. Con group, ^#^*P*< 0.05, ^##^*P*< 0.01 vs. NaT group, ^∆^*P*< 0.05 vs. VB 12 (100 nM) group. Con: control group. NaT: acute pancreatitis group. All the experiments were repeated three times with similar results.


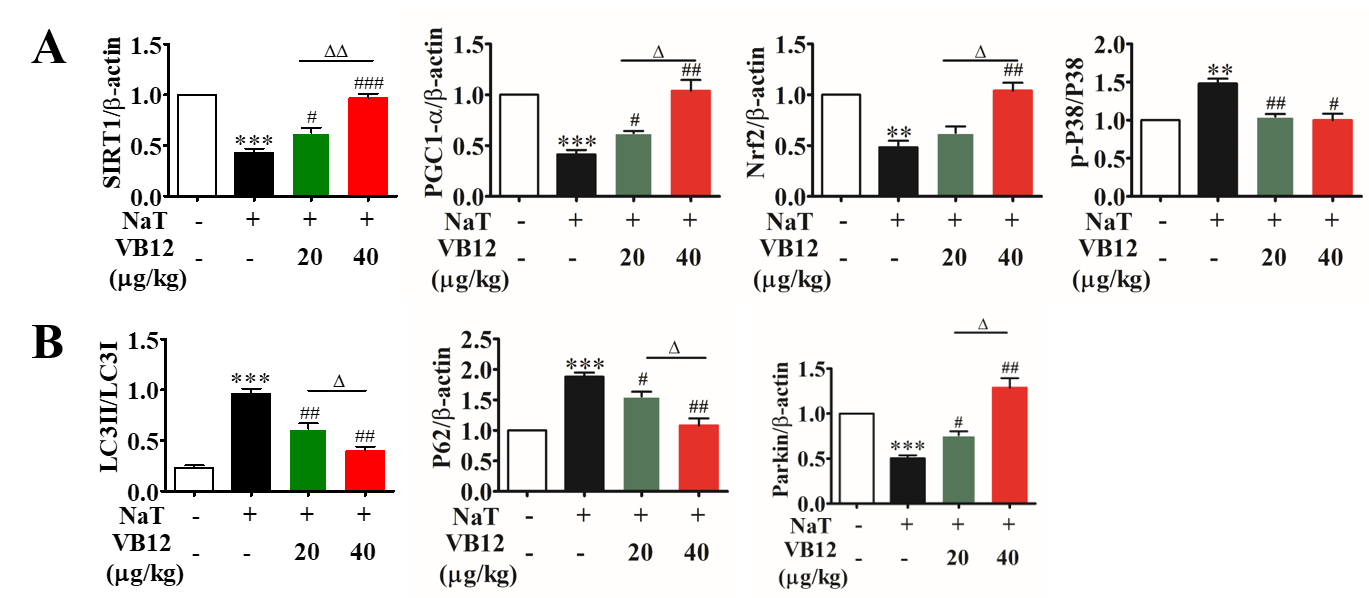


**Fig. S9. Clarify the quantitative results of protein bands in Fig.6.** (A) Densitometry analysis results of SIRT1, PGC1-α, Nrf2, p-P38, P38 proteins. (B) Densitometry analysis results of SQSTM1 (p62), LC3II/LC3I and Parkin proteins. Presented values are means ± SEM. ***P*< 0.01 vs. Con group, ****P*< 0.0001 vs. Con group, ^#^*P*< 0.05, ^##^*P*< 0.01 , ^###^*P*< 0.001 vs. NaT group, ^∆^*P*< 0.05 , ^∆∆^*P*< 0.01 vs. VB 12 (20 μg/kg) group. Con: control group. NaT: acute pancreatitis group. All the experiments were repeated
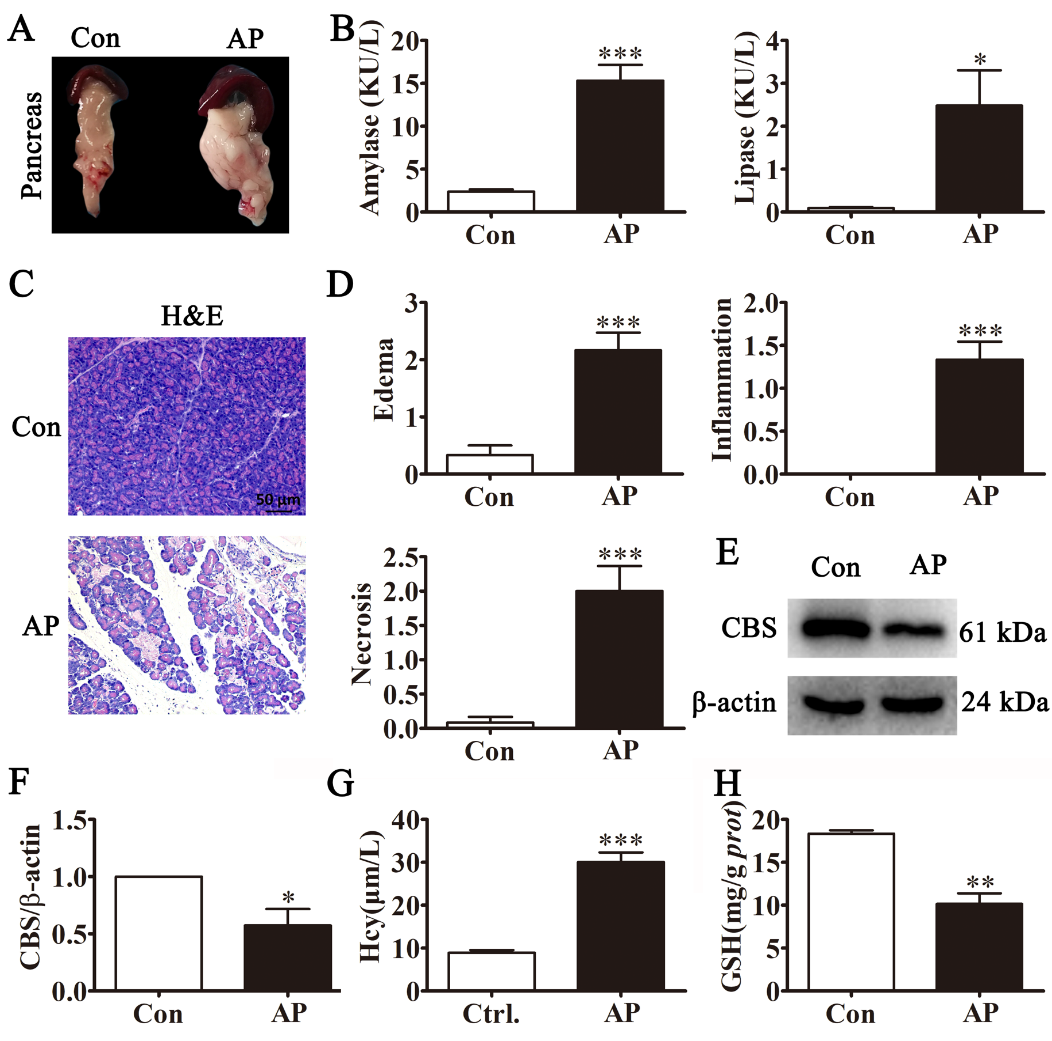
three times with similar results.

**Fig. S10. CBS loss is associated with pancreatic injury in AP mice.** Acute pancreatitis model was established by retrograde pancreatic ductal injection of 3 % NaT (n = 6). (A) The general morphology of the pancreas was observed. (B) The levels of amylase and lipase in serum were detected. (C) Histological damage of pancreas was evaluated by H&E staining (200 ×). (D) Pancreatic histopathology scores including edema, inflammatory cell infiltration and necrosis were evaluated blindly by two pathologists. (E-F) The expression of CBS in pancreatic tissue was detected by western blot. β-actin was used as internal standards. (G) The level of homocysteine in serum was detected by ELISA assay kit. (H) The ratio of GSH / GSSG in mouse pancreas was detected 24 hours after NaT treatment. The results are presented as the mean ± SEM. ****P* < 0.001 vs. Con group. **P* < 0.05 vs. Con group. Con: control group. AP: acute pancreatitis group.


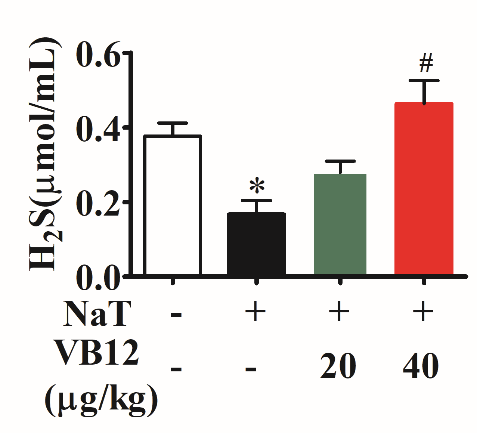


**Fig. S11. VB 12 increases the content of H_2_S in serum.** Animals were randomly divided into four experimental groups: AP group treated with NaT (3 %), NaT + VB 12 (20 μg/kg, ip), NaT + VB 12 (40 μg/kg, ip) and control group treated with 0.9 % saline (n = 6). NaT (3 %) was injected through the pancreaticobiliary tract, and blood was collected at 24 hours for follow-up studies. The content of H_2_S in serum was detected by H_2_S Assay Kit. The results are presented as the mean ± SEM. **P* < 0.05 vs. Con group. ^#^*P* < 0.05 vs. NaT group. Con: control group. NaT: acute pancreatitis group.
